# Optimizing tumor-associated antigen-stimulated autologous dendritic cell and cytokine-induced killer cell coculture to enhance cytotoxicity for cancer immunotherapy in manufacturing

**DOI:** 10.1186/s12865-023-00552-5

**Published:** 2023-06-29

**Authors:** Yi-Yen Lee, Shao-Ciao Luo, Chung-Hsin Lee, Chien-Lun Tang, Chiung-Chyi Shen, Wen-Yu Cheng, Yi-Chin Yang, Meng-Yin Yang, Chun-Ming Yen

**Affiliations:** 1 Division of Pediatric Neurosurgery, Department of Neurosurgery, Neurological Institute, Taipei Veterans General Hospital, Taipei, Taiwan; 2 School of Medicine, National Yang Ming Chiao Tung University, Taipei, Taiwan; 3 Departments of Surgery, Taichung Veterans General Hospital, Taichung, Taiwan; 4 Department of Neurosurgery, Neurological Institute, Taichung Veterans General Hospital, Taichung, Taiwan; 5 School of Medicine, National Defense Medical Center, Taipei, Taiwan; 6 Basic Medical Education Center, Central Taiwan University of Science and Technology, Taichung, Taiwan; 7 Department of Physical Therapy, Hung Kuang University, Taichung, Taiwan; 8 Institute of Biomedical Sciences, National Chung Hsing University, Taichung, Taiwan; 9 Department of Post-Baccalaureate Medicine, College of Medicine, National Chung Hsing University, Taichung, Taiwan

**Keywords:** Dendritic cells, Cytokine-induced killer cells, Tumor-associated antigen, Cytotoxic cell, Peripheral blood monocyte

## Abstract

**Background:**

Dendritic Cell Cytokine-induced killer cell (DC-CIK) coculture treatment in cancer immunotherapy has been shown to be effective. However, the cost of DC- CIK therapy is prohibitive for many patients, and the lack of standard manufacturing processes and treatment strategies are major limitations. Our study used tumor lysate as a tumor-associated antigen source and DCs and CIK cells in coculture. We developed an efficient method to obtain autologous DCs- and CIK cells from peripheral blood. We used flow cytometry to assess DC activation and the cytometric bead array assay to quantify cytokines secreted by CIK cells.

**Results:**

We evaluated the antitumor activity of DC- CIK coculture in vitro with the K562 cell line. We demonstrated that a manufacturing process employing frozen immature DCs can yield the lowest loss with the highest economic benefits. DC-CIK coculture can effectively upgrade CIK cells’ immunological specificity to tumors in the presence of tumor-associated antigens.

**Conclusion:**

In vitro experiments revealed that when the DC- CIK cell ratio was 1: 20 in the coculture, CIK cells secreted the highest number of cytokines on the 14th day and the antitumor immune effect showed the highest potency. CIK cells’ cytotoxicity to K562 cells was highest when the CIK: K562 cell ratio was 25: 1. We developed an efficient manufacturing process for DC- CIK coculture, while also establishing the optimal DC- CIK cell ratio for immunological activity and the best cytotoxic CIK: K562 cell ratio.

## Background

Immunotherapy combined with surgery, radiation therapy, chemotherapy, or targeted therapy has become one of the most common forms of cancer treatment. It includes immune checkpoint blockade, adoptive cell therapy, and cancer vaccines [1]. Immune checkpoint blockade involves targeting the PD-1/PD-L1 and CD80/CTLA-4 interactions [1]. Adoptive cell therapy deploys tumor-infiltrating lymphocytes, cytokine-induced killer (CIK) cells, and chimeric antigen receptor T-cells to destroy tumors [2]. Cancer vaccines include prophylactic vaccines, such as the hepatitis B virus and human papillomavirus vaccines, and therapeutic vaccines, such as the bacilli Calmette-Guerin vaccine as well as various new personalized recombinant cancer vaccines [3].

CIK cells are cytotoxic T lymphocytes regarded as the most cytotoxic immune cells. They are produced by ex-vivo culturing of peripheral lymphocytes in a cytokine cocktail containing CD3 monoclonal antibodies, interleukin (IL), and interferon (IFN) [4]. Enhanced Cytotoxic Activity and Potent Killing Properties of the Cocktail in both In Vitro and In Vivo Experiments [5–8]. CIK cells’ anticancer potential and ability to prolong survival were first reported in a mouse model with severe combined immunodeficiency and a human lymphoma model [9]. CIK therapy offers cancer patients the advantages of high efficiency, low toxicity, prolonged overall survival, and disease-free survival [10–12].

Dendritic cells (DCs) are potent antigen-presenting cells in the human body and were first used to develop anticancer vaccines in the mid-1990s [13]. In recent years, the emergence of immune checkpoint inhibitors and genetically modified cell-based platforms in the cancer immunotherapy landscape has rekindled interest in DC-based therapies.

DCs play a critical role in activating immune responses by facilitating the generation of both helper and cytotoxic T cells, as well as presenting antigens to T lymphocytes [14, 15]. In the context of cancer immunotherapy, DCs are co-cultured and educated to recognize tumor-associated antigens, thereby serving as antigen-presenting cells to activate the immune system. After activation, T cells begin to move toward the peripheral circulation, searching for cells that carry tumor-associated antigens and then attacking the tumor with these specific antigens. The innate properties of DCs in antigen presentation can effectively counteract the lack of specificity of CIK cells and enhance their cytotoxicity [16]. Therefore, the combination of DCs and CIK cells can increase the antitumor immune response and boost cytotoxicity to tumor cells.

Currently, the lack of a certified standard process for manufacturing the DC- CIK cell coculture (DC- CIK) immunotherapy and its prohibitive cost is the primary challenges for patients wishing to undergo this treatment. Our study demonstrates the most economical manufacturing process for DC- CIK, while also establishing a therapeutic model with the highest cytotoxic effect.

## Methods

### Preparation of tumor-associated antigens

#### Patients

This study was approved by the Institutional Review Board of Taichung Veterans General Hospital, Taichung, Taiwan (approval code: CF21345B). Liver tumor samples were obtained by surgical resection of hepatocellular carcinoma of patients diagnosed based on clinical and histopathological criteria. A blood sample was also obtained during the resection. All participants were enrolled in this study in accordance with the Declaration of Helsinki principles for research involving human subjects. Consent was obtained from all participants before clinical information and blood samples were collected.

Tumor cells from each specimen were cultured in a dish containing Gibco^™^ IMDM (Iscove’s modified Dulbecco’s medium) for six to eight weeks as the source of antigens. The tumor cells were subjected to five cycles of freezing- and thawing to destroy the cells and release antigens. In the freezing step, the specimen was placed in a cryovial; then, the mouth of the cryovial was sealed with a heat-sealing film and the vial was directly immersed in liquid nitrogen for 10 minutes. In the thawing step, the vial was placed in a water bath for 10 minutes at 37°C. The processed sample was centrifuged and the supernatant was filtered with a 0.22 µm PVDF filter (Merck Millipore) to obtain the tumor lysate, which contained tumor antigens. A part of the lysate was cultured in a T25 flask for approximately four weeks in order to confirm that there were no residual live tumor cells. Indeed, iDCs in our study were stimulated using tumor lysate rather than intact tumor cells. To prepare the tumor lysate, tumor cells were subjected to multiple cycles of freezing and thawing. This process effectively disrupted the cells and facilitated the release of antigens. By utilizing tumor lysate as the stimulus, we aimed to expose iDCs to a broad range of tumor antigens, allowing for a comprehensive activation of the immune response.

### Preparation of iDC, mDC, and CIK cells

At least 750 ml of peripheral blood was taken from each patient for leukapheresis. Then, peripheral blood mononuclear cells (PBMCs) were isolated using a Ficoll separation solution. The PBMCs were allowed to adhere for two hours and the non-adherent cells were washed away. The adherent cells were incubated in TrypLE^™^ cell-dissociation enzyme (Gibco^™^) and cultured in Lonza Xvivo-15 medium. The cytokines GM-CSF (500U/ml, CellGenix®) and IL-4 (1000U/ml, CellGenix®) were added to the medium to induce differentiation of PBMCs into immature DCs (iDCs) over 5–6 days before the cells were frozen. The frozen iDCs were thawed afterward and cultured with cytokine or antigen to promote the iDCs to develop into mature DCs (mDCs).

Furthermore, prior to each injection requiring CIK cells, each patient was provided with 30 ml of peripheral blood two weeks in advance. Peripheral blood mononuclear cells (PBMCs) were isolated following leukapheresis, and ex vivo generation of CIK cells was carried out by incubating the PBMCs with anti-CD3 antibodies, IL-2, and IFN-γ. This incubation process involved the treatment of cells with anti-CD3 antibodies in the presence of IL-2 and IFN-γ, which facilitated their expansion and activation.

Each of the above steps involved mycoplasma detection and the broth test, as well as the Limulus amebocyte lysate test and analysis of Gram stain, flow, cell number, bicinchoninic acid assay for protein, T25 seeding, and other tasks at different stages. The above production process for DC met the standards published in a previous article [17].

### Preparation of cytokine-stimulated DC- CIK

DC–CIK was produced after coculturing mDCs and CIK cells. mDCs were first developed by stimulation of iDCs over 48 hours with the cytokines TNF-α (2000 U/ml, R&D) and IL-1β (2000 U/ml, R&D). Then, the cytokine-stimulated mDCs were cocultured with 20 times and 100 times the number of CIK cells. On the 9th and 14th day, the cytokine-stimulated DC–CIK was analyzed using flow assay, cell cytotoxicity assay with K562 cells, and cytometric bead array (CBA) assay.

The differences in cell markers, cytokines, proportion, the number and viability of CIK cells, and the K562 cell-killing rate were compared between CIK cells only (the control) and DC- CIK at the DC: CIK cell ratios of 1: 20 and 1: 100 on the 9th and 14th day.

In our study, the total recovery rate of mDC was calculated by determining the percentage of mDCs obtained after the deployment process compared to the initial number of iDCs used for the generation of mDCs. The formula used to calculate the total recovery rate is as follows:

Total Recovery Rate (%) = (Number of mDCs obtained after deployment / Number of iDCs used for mDC generation) x 100

The number of mDCs obtained after deployment was determined by counting the viable mDCs using a hemocytometer or flow cytometry, depending on the specific experimental setup. The number of iDCs used for mDC generation refers to the initial number of iDCs that underwent the deployment process.

### Preparation of Ag + cytokine-stimulated DC- CIK

Following 24 hours of antigen stimulation of iDCs using K562 (cell concentration of 0.3–0.5 × 10^6^/ml in GibcoTM IMDM), the stimulation process was extended for an additional 24 hours with the addition of the cytokines TNF-α and IL-1β. Subsequently, the Ag + cytokine-stimulated mDCs were co-cultured with a 20-fold and 100-fold increase in the number of CIK cells.

The differences in cell markers, cytokines proportion, the number and viability of CIK cells, and the K562 cell-killing rate were compared between control and DC- CIK at the DC: CIK cell ratios of 1: 20 and 1: 100 on the 9th and 14th day.

### Flow cytometry analysis

To analyze the cells, we prepared PBMCs as a single-cell suspension. The PBMCs were then treated with Fixable Viability Dye eFluorTM 780 on ice for 30 minutes to exclude dead cells. Following that, surface antibodies targeting specific markers including CD86 (clone B7-2, Invitrogen), CD83 (EPR23809-19, Abcam), CD40 (clone 1C10, Invitrogen), CD14 (clone MEM-15, Merck), and HLA-DR (clone LN3, Invitrogen) were added to the cells and incubated on ice for 30 minutes. To account for non-specific binding and background fluorescence, appropriate isotype control antibodies were used during the flow cytometry analysis. These isotype controls served as negative controls to establish baseline fluorescence levels and ensure the specificity of our staining. The cells were then washed several times. For intracellular staining, we employed the FoxP3 Fixation and Permeabilization Kit (eBioscience) to permeabilize the cells. Cell analysis was conducted using a BD FACSCalibur Flow Cytometry System, and the resulting data were analyzed using CellQuest software.

### Cytometric bead array (CBA)

To measure the medium concentrations of IL-10, IL-6, INF-γ, IL-4, and IL-2, we utilized the CBA human Th1/Th2 Cytokine kit II (BD Bioscience), following the manufacturer’s instructions and established protocols described in previous publications [31]. Briefly, the sample was incubated with the capture beads and detection reagents at room temperature for approximately 2 h and 30 min. After a thorough washing step, cytokine concentrations were determined using flow cytometry fluorescence.

### Statistical analysis

The data were presented as mean ± standard error of the mean. Independent experiments were pooled when the coefficient of variance could be assumed identical. Independent t-tests and one-way analysis of variance (ANOVA) were used to analyze the effects of group (CIK vs. DC-CIK) treatment. The results were considered to be of statistical significance with a value of p < 0.05 and p < 0.01.

## Results

### Preparation of tumor lysate, iDC, and mDC

**Fig. 1:**
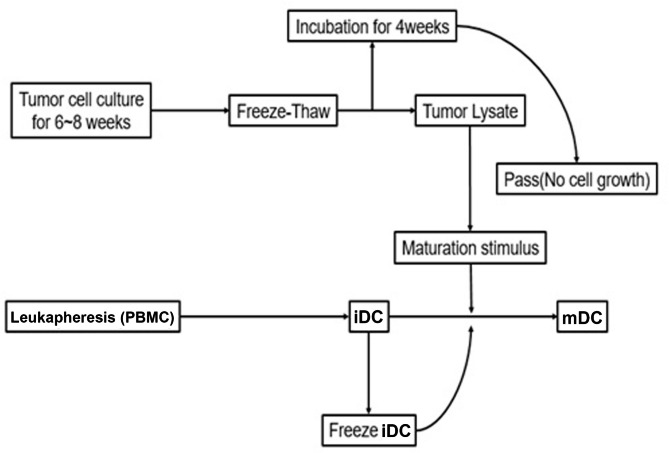
Schematic diagram of the produce protocol for mature dendritic cells (mDCs). The Peripheral blood mononuclear cells (PBMCs) were obtained from leukapheresis of peripheral blood and were cultured to produce immature dendritic cells ( iDCs). Tumor cells were cultured for 6–8 weeks and then subjected to a repeated freeze- thaw process to obtain a tumor lysate to stimulate the development of iDCs into mature dendritic cells (mDCs) that can recognize tumor-associated antigens

Tumor cell culture took 6–8 weeks from the time the tumor specimen was obtained to generate enough cells to produce tumor-associated antigens. The tumor lysate was obtained within one day via the repeated freeze- thaw process, and T25 incubation of the lysate took at least four weeks. The tumor-associated antigens were prepared from the tumor specimen in 10–12 weeks. After the patient’s blood was drawn, leukapheresis was performed to screen for PBMCs. GM-CSF and IL-4 were added to PBMCs for expansion and differentiation into iDCs. This process took approximately 5–6 days. iDCs were converted to mDCs with stimulants (TNF-α or K562-P26, P27 in this experiment) in two days. Therefore, 7–8 days were necessary to obtain autologous mDCs from peripheral blood (Fig. 1).

### Protocol for mDC deployment

In the standard procedure, peripheral blood is drawn once to perform leukapheresis to acquire a large amount of PBMCs as the source of DCs. Considering that the course of treatment may be a phased injection, the cells have to be cryopreserved and thawed when the injection is required. We present the freezing tests performed at different stages of the process in order to obtain the freezing protocol with the lowest loss. Three protocols are described in Table 1.

1. Frozen iDCs: PBMCs from leukapheresis samples were separated using Ficoll isolation and cultured to produce iDCs for freezing. The iDCs were thawed and cultured to produce mDCs before each injection.
2. Frozen PBMCs: PBMCs from leukapheresis samples were separated using Ficoll isolation and directly frozen, thawed, and cultured to produce mDC before each injection.
3. Frozen mDC: PBMCs from leukapheresis samples were separated using Ficoll isolation and cultured to produce mDCs for freezing. The mDCs were thawed before each injection.

**Table 1:** Summary of three manufacturing methods to prepare mature dendritic cell recovery rate

	Thaw recovery rate	PBMC→iDC	iDC→mDC	The total recovery rate (mDC)
portocol 1. fresh PBMC > iDC > F-iDC > mDC	83.90%	7.28%	36.76%	2.25% (*)
protocol 2. freeze PBMC > iDC > mDC	60.77%	4.31%	56.43%	1.91%
portocol 3. PBMC > iDC > mDC > F-mDC > mDC	62.00%	4.10%	79.56%	2.14%

We compared three manufacturing methods’ mature dendritic cell (mDC) recovery rates. The lowest mDC recovery rate of 1.91% was obtained with frozen peripheral blood mononuclear cells. The mDC recovery rate from frozen mDCs was 2.14%. The highest recovery rate was 2.25% with cryopreserved immature dendritic cells (iDCs). * indicates p-value < 0.05 compared with the protocol2 on the Student t-test

We found that the total recovery rate of mDC was 2.25% in protocol 1, 2.14% in protocol 3, and 1.91% in protocol 2. Therefore, we decided to use protocol 1, which had an acceptable recovery rate. The thawed iDCs had to undergo additional incubation to enable them to develop into mature mDCs, but this additional time could be spent on buffering and observing the thawing of the cells in order to facilitate the subsequent cultivation with CIK cells.

### Differences between DC- CIK and the CIK cells only control

**Fig. 2:**
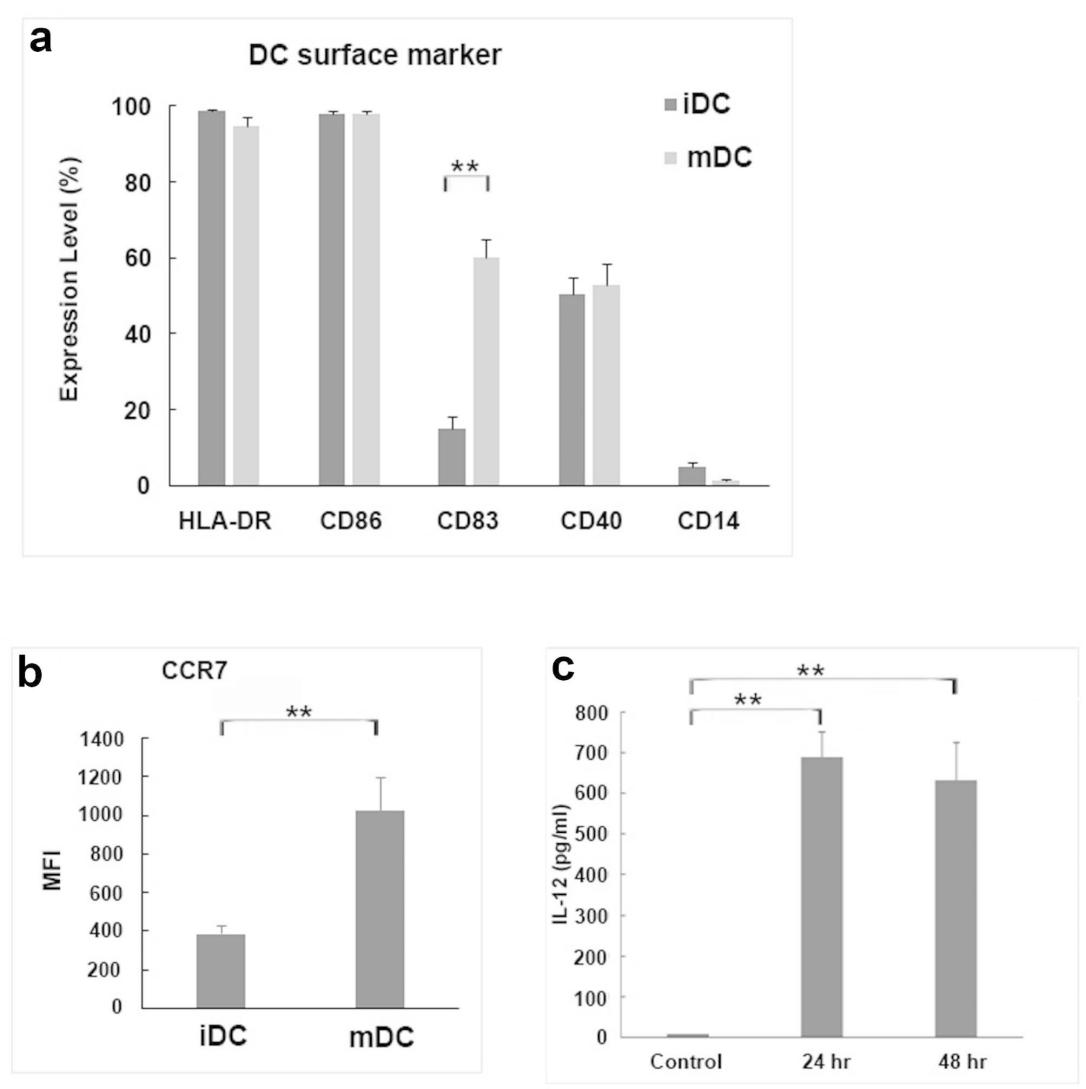
Mature dendritic cells (mDCs) are characterized by marker expression and IL-12 production. (**a**) Quantitative analysis of antigen-presenting cell activation markers. The DC gating of mDCs was slightly lower than that of immature dendritic cells (iDCs). The expression of HLA-DR, CD86, and CD40 markers in iDCs and mDCs is similar. The expression of CD83 and (**b**) CCR7 markers in mDCs was higher than that in iDCs, and the expression of the CD14 marker in mDCs was significantly decreased. (**c**) The amount of IL-12 measured at the end of DC maturation showed an increase in production capacity. ** indicates p < 0.01. The results were repeated over three independent experiments in each case

Differences between DC-CIK and CIK cells only control were evaluated through quantitative flow cytometry analysis during DC production. On the fifth day (D5), 48.0% of the total analyzed cells differentiated into immature DCs (iDC), while on the seventh day (D7), 44.4% of the cells matured into mature DCs (mDC) (Fig. 2a). To enhance the stimulatory capacity of DCs, we established specific criteria for assessing their maturation, including HLA-DR > 70%, CD86 > 70%, and CD14 < 20%. Furthermore, we investigated the CD40L-induced IL-12 production from iDCs and mDCs. Flow cytometry analysis encompassed the measurement of HLA-DR, CD86, CD83, CD40, CD14, and CCR7, serving as markers for DC phenotypes and activation of antigen-presenting cells, indicative of mDC maturation. Notably, mDC cells exhibited increased expression of CCR7 (Fig. 2b), and their high IL-12 production capacity further confirmed their maturation state (Fig. 2c). It is important to clarify that the “48.0%” mentioned in the sentence refers to the percentage of cells that differentiated into iDC (immature dendritic cells) among the total cells analyzed in the flow cytometry analysis.

**Fig. 3:**
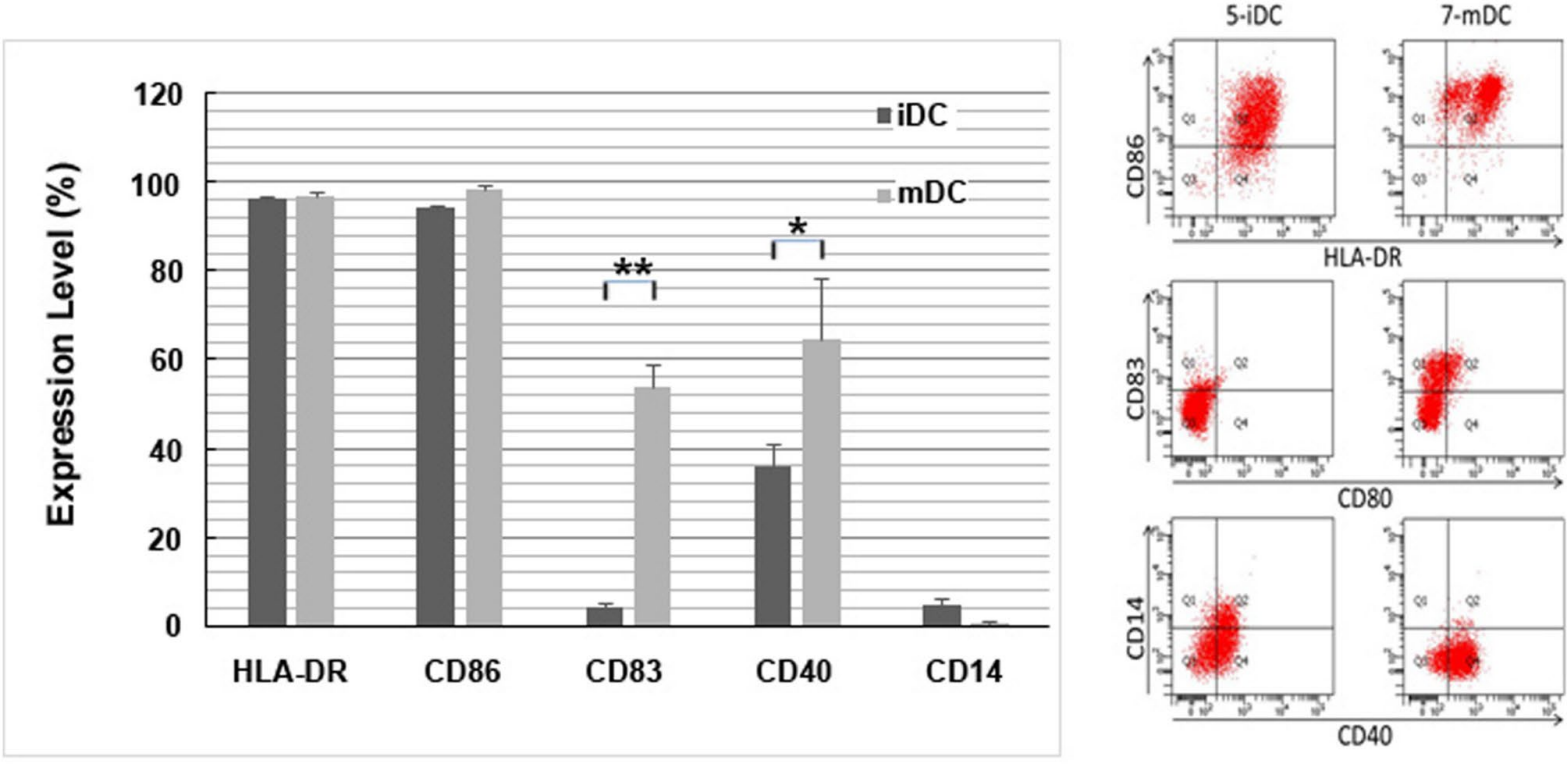
The comprehensive evidence of the impact of TNF-α and IL-1β stimulation on dendritic cell (DC) maturation, as demonstrated through quantitative analysis and flow cytometry. The data clearly show a significant upregulation of these molecules’ expression levels on both iDCs and mDCs following TNF-α and IL-1β stimulation, underscoring their crucial role in promoting DC maturation. The values presented are the mean ± SEM results obtained from three independent experiments. Significance levels are indicated as * for p < 0.05 and ** for p < 0.01

Cytokines (TNF-α + IL-1β) were added to stimulate iDCs to mature into mDCs. For the DC phenotype, flow cytometry measurements were obtained for HLA-DR, CD86, CD80, CD83, CD40, and CD14, which represented antigen-presenting cell activation markers. This implies that the addition of cytokines (TNF-α + IL-1β) can promote the maturation of DCs (Fig. 3).

CIK cells are mainly expanded T lymphocytes in vitro. Therefore, they exhibit a large number of CD3 markers. We compared the differences in activated marker expression, cytotoxicity to K562 cells, and the amount of CIK-secreted cytokines between DC- CIK cells and the control (i.e., CIK cells only). In the comparison between the control and cytokine-stimulated DC- CIK on the 9th day of DC: CIK coculture at a ratio of 1: 20, CIK cells expression of CD56, CD3-/CD56, CD4, CD8, CD28, etc. showed no differences. The cytotoxicity rate towards K562 cells in the cytokine-stimulated DC-CIK group was consistently lower compared to the control group, regardless of the CIK:K562 cell ratio (25:1, 5:1, or 1:1). This showed that cytokine-stimulated DC- CIK did not influence activated marker expression and that their cytotoxicity to K562 cells was lower. When the DC: CIK cell ratio was increased to 1: 100 marker expression of cytokine-stimulated DC- CIK was not elevated on the 9th day compared to that of the control. Instead, the rate of cytotoxicity to K562 cells of cytokine-stimulated DC- CIK was lower than that of the control, irrespective of the CIK: K562 cell ratio.

**Fig. 4:**
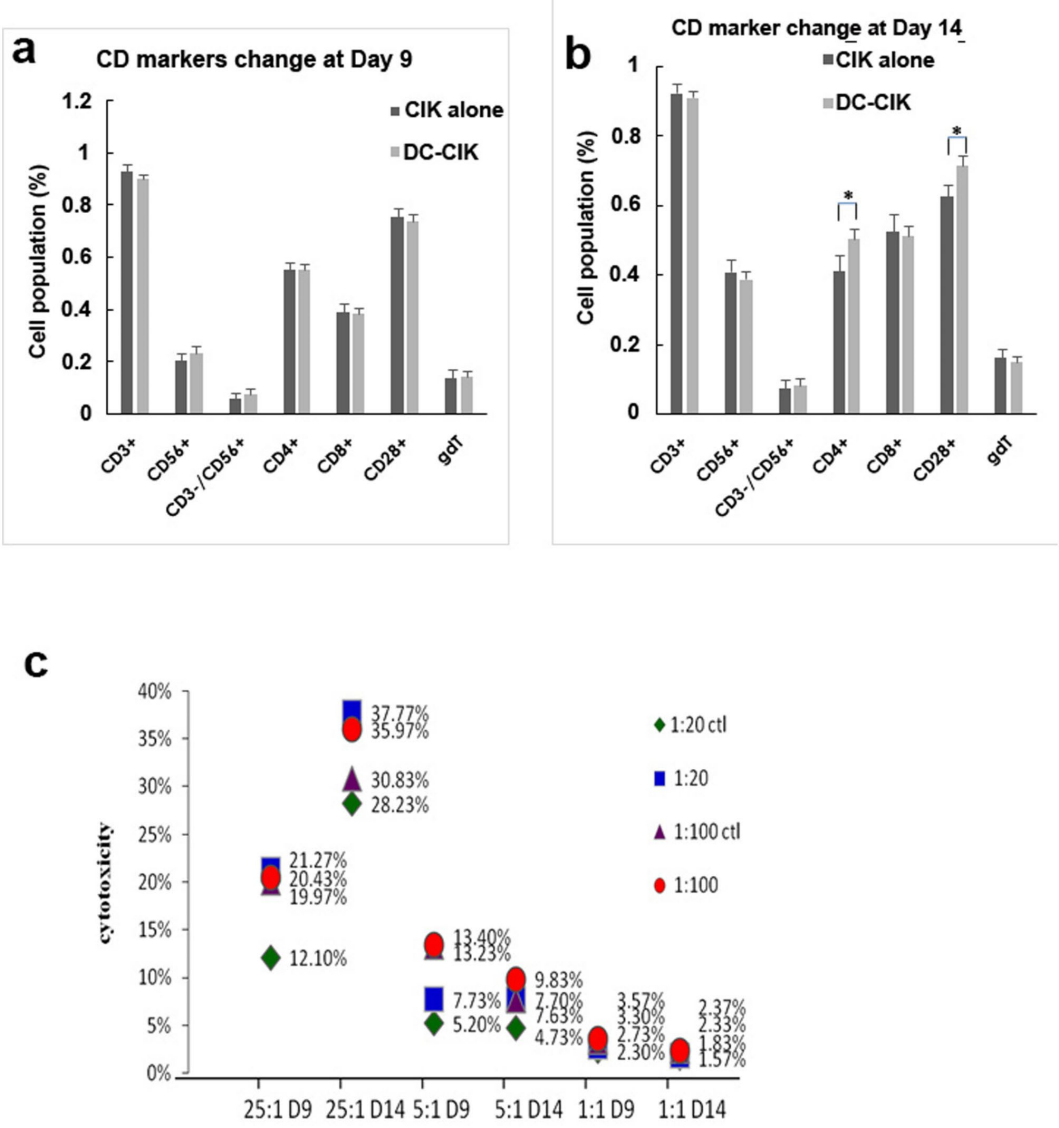
Comparison of cell marker expression and cytotoxicity (**a**) Comparison of cytokine-induced killer (CIK) cell marker expression on the 9th and 14th day between cytokine-stimulated dendritic cell (DC)- CIK cell coculture different DC: CIK cell ratios and the CIK cells-only control. (**b**) Comparison of cytotoxicity between cytokine-stimulated DC- CIK cell coculture and the control (CIK alone) at different CIK: K562 cell ratios on the 9th and 14th day. * indicates p-value < 0.05 compared with the control on the Student t-test. (**c**) We compared the cell numbers and viability between the antigen with cytokine co-stimulated DC-CIK group and the control group at different ratios of DC:CIK on the 9th and 14th days

On the 14th day, the expression of CIK cell markers CD4 and CD28 was higher in cytokine-stimulated DC- CIK at the DC: CIK ratio of 1: 20 than in the control (CIK alone). When the DC: CIK ratio was 1: 100, the expression of CIK cell markers CD4 and CD28 increased more in cytokine-stimulated DC- CIK than in the control. However, there was no difference between cytokine-stimulated DC- CIK and the control in the rate of cytotoxicity to K562 cells, regardless of whether the CIK: K562 ratio was 25: 1 or 5: 1 (Fig. 4).

On the 9th day, INF-γ and IL-2 secretion by cytokine-stimulated DC- CIK was elevated at the DC: CIK cell ratio of 1: 20, while TNF-α, IL-10, IL-6, and IL-4 secretion was reduced, compared to the control. On the 14th day, the secretion of cytokines of IFN-γ, TNF-α, IL-10, IL-6, and IL-4 by CIK cells was elevated in cytokine-stimulated DC- CIK compared to the control. On the 9th day, the number and viability of CIK cells were the same for cytokine-stimulated DC- CIK and the control, but on the 14th day, CIK cell viability increased and cell numbers decreased in the former.

**Fig. 5:**
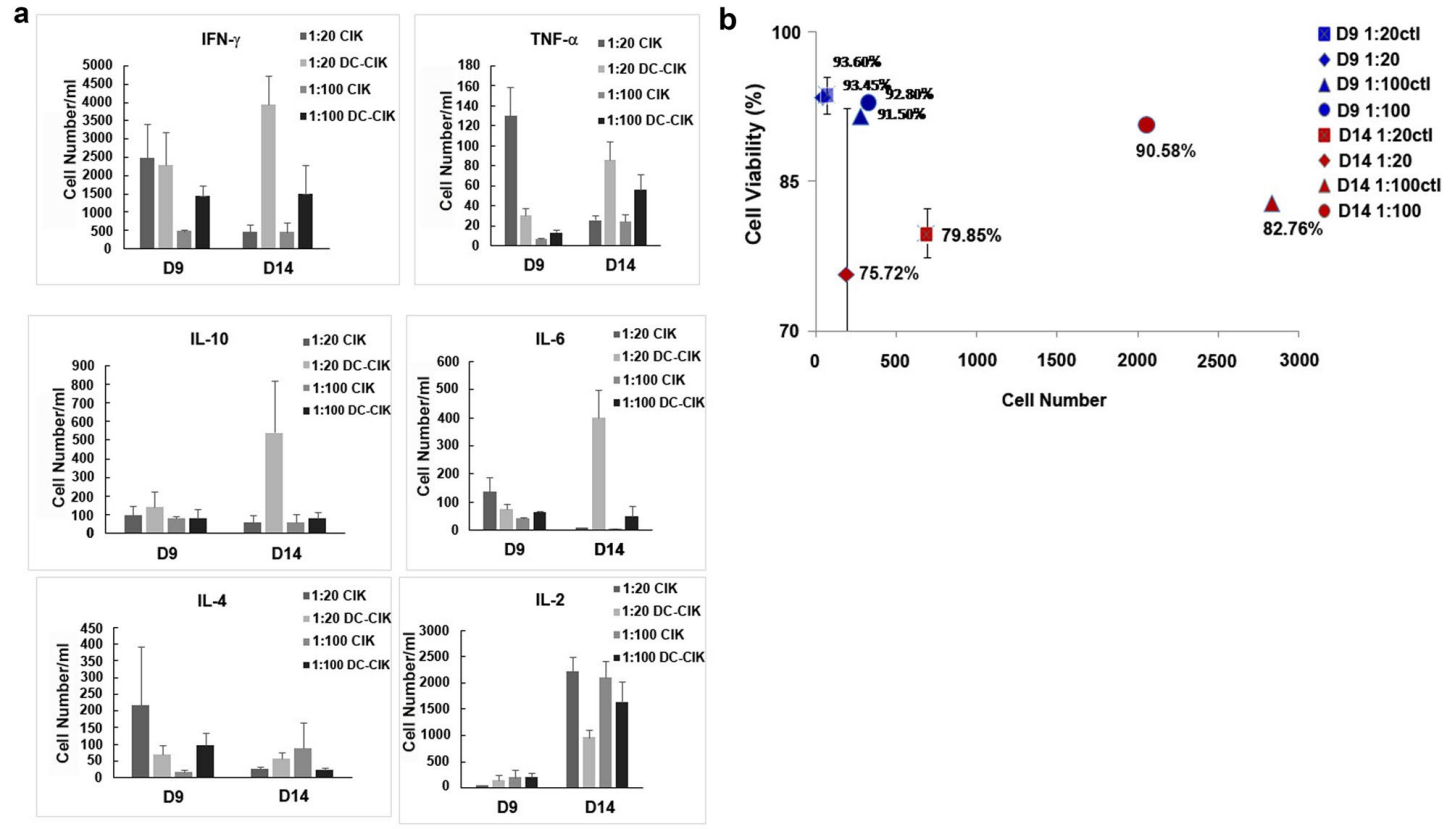
Comparison of cell ratio and viability. (**a**) Comparison of the number of cytokines secreted by cytokine-induced killer (CIK) cells at different dendritic cell (DC): CIK cell ratios between cytokine-stimulated DC- CIK cell coculture and control on the 9th and 14th day. (**b**) Comparison of cell number and viability between cytokine-stimulated DC- CIK cell coculture and the control at different DC: CIK cell ratios on the 9th and 14th day. The values presented are the mean ± SEM results obtained from three independent experiments. Significance levels are indicated as * for p < 0.05 and ** for p < 0.01

The DC:CIK cell ratio was adjusted to 1:100. On the 9th day, cytokine-stimulated DC-CIK exhibited increased secretion of IFN-γ, TNF-α, IL-10, IL-6, and IL-4 by CIK cells, except for IL-2, compared to the control group. By the 14th day, cytokine-stimulated DC-CIK showed increased secretion of IFN-γ, TNF-α, IL-10, IL-6, and IL-4, except for IL-2 secretion. The number and viability of CIK cells did not significantly differ between cytokine-stimulated DC-CIK and the control on the 9th day. However, on the 14th day, the cell numbers were lower, although the viability was higher in cytokine-stimulated DC-CIK compared to the control (Fig. 5). It is important to note that the cytokines analyzed in our study were measured from the culture supernatant of the DC and CIK coculture.

### Differences between antigen-stimulated DC- CIK and the CIK cells-only control

An antigen (K562-P26, P27) was used to stimulate iDCs for 24 hours, and then cytokines (TNF-α + IL-1β) were used for another 24 hours to stimulate iDCs to mature into mDCs. Flow cytometry measured the DC phenotype which showed activated markers of antigen-presenting cells, including HLA-DR, CD86, CD80, CD83, CD40, and CD14. mDCs showed increased expression of these markers, except for CD14 that showed a decrease, on the 7th day compared to marker expression in iDCs on the 5th day, indicating that antigen (K562-P26, P27) combined with cytokines (TNF-α + IL-1β) can stimulate DC maturation (Fig. 2).

We compared the differences in marker expression, cytotoxicity to K562 cells, and the amount of CIK-secreted cytokines between antigen-stimulated DC- CIK and the control. On the 9th day of the 1: 20 DC: CIK coculture, the expression of cell markers CD56, CD3-/CD56, CD4, CD8, and CD28 was not different between Ag + cytokine-stimulated DC-CIK and the control. However, the K562 cell-killing rate of CIK cells increased in Ag + cytokine-stimulated DC- CIK, irrespective of whether the CIK: K562 cell ratio was 25: 1, 5: 1, or 1: 1. The most significant increase in the rate, 21.27%, occurred at the CIK: K562 cell ratio of 25: 1. On the 14th day, the expression of CIK markers, including CD4 and CD28, showed a higher increase in Ag + cytokine-stimulated DC- CIK than in the control. Cytotoxicity to K562 cells also increased, regardless of whether the CIK: K562 cell ratio was 25: 1, 5: 1, or 1: 1. At the CIK: K562 cell ratio of 25: 1, the rate of cytotoxicity showed the most significant change of an increased by up to 37.77%.

The DC: CIK cell ratio was adjusted to 1: 100. The expression of CIK markers, CD56, CD3-/CD56, CD4, CD8, and CD28, was similar in Ag + cytokine-stimulated DC-CIK and control on day 9. Only CD4 and CD28 increased on day 14 in Ag + cytokine-stimulated DC- CIK compared to the control. The K562 cell-killing rate of Ag + cytokine-stimulated was similar to that of the control on the 9th day, irrespective of whether the CIK: K562 cell ratio was 25: 1, 5: 1, or 1: 1. On the 14th day, the K562 cell-killing rate of Ag + cytokine-stimulated DC- CIK increased compared to that of the control at the CIK: K562 cell ratio of 25: 1 and 5: 1, but was similar at the 1: 1 ratio. At the DC: CIK cell ratio of 1: 100 and CIK: K562 cell ratio of 25: 1, the rate of cytotoxicity increased up to 35.97%, which was close to 37.77% at the DC: CIK cell ratio of 1: 20 together with the CIK: K562 cell ratio of 25: 1. However, the more cells are used, the higher the cost. We found that on the 14th day, when the DC: CIK cell ratio was 1: 20 and the CIK: K562 cell ratio was 25: 1, the K562 cell-killing rate of CIK cells was up to 37.77%, which was the most significant and economical rate (Fig. 4).

Regardless of whether the DC: CIK cell coculture ratio was 1: 20 or 1: 100, on the 9th day, the amount of cytokines IFN-γ, TNF-α, IL-10, IL-6, and IL-4 secreted by Ag + cytokine-stimulated DC- CIK increased compared to the control. At the DC: CIK cell ratio of 1: 20, the amount of IFN-γ, TNF-a, IL-10, IL-6, and IL-4 secreted by Ag + cytokine-stimulated DC- CIK increased on the 14th day compared to the control. At the DC- CIK cell ratio of 1: 100, the amount of INF-γ, IL-10, and IL-6 secreted by Ag + cytokine-stimulated DC- CIK was higher but the amount of TNF-a, IL-4, and IL-2 secreted were lower compared to the control. We also found that on the 14th day, the number of cytokines secreted by Ag + cytokine-stimulated DC-CIK cell ratio of 1: 20 than at the ratio of 1: 100.

Cell viability on the 14th day was lower than that on the 9th day, regardless of whether the DC: CIK cell ratio was 1: 20 or 1: 100, indicating that cell viability declined over time. On the 9th day, the number of CIK cells in Ag + cytokine-stimulated DC- CIK was similar to that in the control regardless of whether the DC: CIK cell ratio was 1: 20 or 1: 100. On the 14th day, at the DC: CIK cell ratio of 1: 20, the number of CIK cells in Ag + cytokine-stimulated DC- CIK was lower than that in the control. At the DC: CIK cell ratio of 1: 100, the number of CIK cells in Ag + cytokine-stimulated DC- CIK was slightly higher than that in the control. However, cytokine secretion by CIK cells was at its peak at the DC: CIK cell ratio of 1: 20 on the 14th day, indicating the highest ratio of effecter T cells (Fig. 5). It is important to highlight that in our study, the cytokines analyzed were measured specifically from the culture supernatant of the DC and CIK coculture. The cytokine secretion from DC alone was excluded from the analysis to ensure an accurate interpretation of the results.

## Discussion

The tumor and its surrounding immune cells, fibroblasts, blood vessels, and the extracellular matrix form the so-called tumor microenvironment (TME). The abundant growth factors signaling molecules in the TME and its immunosuppressive state promote tumor progression, invasion, and metastases [18, 19]. The development of cancer immunotherapy depends on understanding the TME. Cancer immunotherapy is mainly provided to overcome the immunosuppressive state of the TME and prevent immune escape by tumor cells. There are currently three main types of cancer immunotherapy: immune checkpoint blockade; adoptive cellular therapies; and cancer vaccines [20]. The role played by the immune system in fighting cancer in humans largely depends on cellular immunity. Adoptive cellular therapy is mainly based on immunological effector T cells. CIK cell therapy is an adoptive cellular therapy. CIK cells are T lymphocytes produced ex vivo and are regarded as having strong cytotoxicity. They mainly work by secreting large amounts of IFN-γ and TNF-α for direct cytotoxic activity, and can also secrete antitumor cytokines such as IL-2 and IL-4, as well as cytokines such as IL-10 and TGF-β, which can antagonize the immune response [21].

DCs are the most important antigen-presenting cells in the human body. They can process antigens and present themselves to T cells to initiate an immune response. There are many types of DC vaccines with antitumor effects [22]. Coculture DCs and CIK can yield a stronger immune response and antitumor effect. Our study showed that cytokines can stimulate the maturation of DCs. However, further analysis found that only cytokines were used in the maturation of DCs when antigen stimulation was lacking; when cytokines stimulated DCs cocultured with CIK cells on the 9th day, the expression of activated markers in CIK cells was unchanged and the K562 cell-killing rate was low. When cytokines stimulated DC- CIK on the 14th day, although activated marker expression in CIK cells increased, the overall K562 cell-killing rate did not increase in concert. This observation demonstrates the cytotoxic effect of CIK cells on K562 cells on day 14, as evidenced by the observed 30–40% killing at an effector-to-target (E:T) ratio of 25:1 [23].

The T cells’ own clone-specific protein complex on the cell surface is known as the T cell receptor (TCR). Most TCRs are composed of α- and β-glycoprotein chains. T cells containing this TCR structure are known as αβ T cells and constitute the majority of conventional T lymphocytes, although a small number of TCRs, those in γδT cells are composed of γ- and δ-chains [24, 25]. Our study found that DC- CIK has a large number of conventional T lymphocyte characteristics including CD3, CD56, CD3-/CD56, CD4, CD8, and CD28 markers, and also contains a small number of γδT cells. We used the CBA assay to analyze the cytokines secreted by CIK cells and found that IFN-γ was the most abundant. In the absence of antigen stimulation, DC- CIK is activated only by cytokines, including IFN-γ and TNF-α that increase over time. However, both an increase in IL-10 and a decrease in IL-2 and IL-4 reduce antitumor immunity and lower the K562 cell-killing rate. IL-6 secretion increases over time, indicating a strong inflammatory response. In addition, we also found that DC- CIK was only activated by cytokines without antigen stimulation, and although cytokines can promote cell viability, the decrease in the total cell number reduced the killing rate. Antigens and cytokines simultaneously stimulated DCs and could induce further DC maturation compared to only cytokine stimulation. When antigen-loaded DCs were combined with CIK cells in cocultured, the activation rate of CIK cells was highest when DC: CIK cells ratio was 1: 20 on the 14th day, although cell number and viability were lower than those when the DC: CIK cell ratio was 1: 100. However, the highest number of cytokines secreted by CIK cells was observed when the DC:CIK cell ratio was 1:20. We further compared the K562 cell-killing rate at different CIK:K562 cell ratios, including 25:1, 5:1, and 1:1, and found that the ratio of 25:1 exhibited the highest killing rate of K562 cells. While it is possible that the total cell number indirectly influenced the overall efficiency of the killing process, it should not have directly impacted the killing rate at the specific effector-to-target (E:T) ratio employed in the study.

However, it is important to acknowledge that our study has certain limitations that require attention. While we have obtained data on IL-12 production from iDCs/mDCs induced by CD40L stimulation, as presented in Fig. 2c, the specific contribution of CD40L stimulus to the observed IL-12 production has not been definitively confirmed.

To address this issue, further investigations and experimental techniques are warranted to establish a direct link between CD40L stimulus and IL-12 production. Additional assays, such as the use of CD40L blocking or specific inhibitors, can be employed to elucidate the precise role of CD40L in inducing IL-12 production. By conducting these supplementary experiments, we aim to enhance our understanding of the relationship between CD40L stimulus and IL-12 production in the context of our study.

### Optimized protocol

We developed an efficient manufacturing process for DC-CIK coculture and determined the optimal ratios of DC-CIK cells and CIK-K562 cells to achieve enhanced immunological activity and cytotoxicity. Based on our study, we recommend the following parameters:

DC Preparation Method: Utilization of frozen immature DCs

1. DC-CIK Cell Ratio: 1:20.
2. CIK-K562 Cell Ratio: 25:1.

Implementing these specific parameters and cell ratios in research and clinical settings can serve as a valuable guide to maximize immunological activity and cytotoxicity outcomes.

## Conclusions

We demonstrated that the production of mDCs from frozen iDCs in an effective formulation with the lowest loss in terms of economic benefits. According to our experimental results, when antigen-loaded DCs are cocultured with CIK cells at a cell number ratio of 1: 20 and harvested on the 14th day, the cytokines secreted reach a peak and show their strongest antitumor response. The CIK cell: tumor cell ratio of 25: 1 yields the highest cytotoxicity. We intend to translate the results of this in vitro experiment and provide data to our clinic so that they can benefit more patients who are seeking access to efficient cancer immunotherapy.

## Data Availability

The datasets utilized and analyzed during the current study are available from the corresponding author upon reasonable request.

## Abbreviations

Ag+: Antigen
CBA: Cytometric bead array
DC-CIK: Dendritic Cell Cytokine-induced killer cell
DCs: Dendritic Cells
GM-CSF: Granulocyte-macrophage colony-stimulating factor
iDC: Immature DCs
IFN: Interferon
IL: Interleukin
mDC: Mature DCs
PBMC: Peripheral blood mononuclear cells
PVDF: Polyvinylidene difluoride
TEM: Tumor microenvironment
